# Sake yeast induces the sleep-promoting effects under the stress-induced acute insomnia in mice

**DOI:** 10.1038/s41598-021-00271-0

**Published:** 2021-10-21

**Authors:** Shohei Nishimon, Noriaki Sakai, Seiji Nishino

**Affiliations:** 1 Sleep and Circadian Neurobiology Laboratory, Department of Psychiatry and Behavioral Sciences, Stanford University School of Medicine, Palo Alto, CA USA; 2 Department of Psychiatry and Behavioral Science, Juntendo University Graduate School of Medicine, Tokyo, Japan; 3 Sleep and Circadian Neurobiology Laboratory, Department of Psychiatry and Behavioral Sciences, Stanford University School of Medicine, 3155 Porter Drive, Rm2106, Palo Alto, CA 94304 USA

**Keywords:** Non-REM sleep, REM sleep, Sleep, Sleep deprivation

## Abstract

Sleep deprivation induces adverse effects on the health, productivity, and performance. The individuals who could not get enough sleep temporarily experience the symptoms of an induced acute insomnia. This study investigated the efficacy of sake yeast in treatment of acute insomnia in mice. The results of this study showed that sake yeast induced a significant dose-dependent wake reduction, a rapid eye movement (REM) and a non-REM (NREM) sleep enhancement during the first 6 h after the oral administration of sake yeast with locomotor activity and core body temperature decreases under the stressful environment in a new cage. In fact, the wake amounts at 3 h and 6 h were significantly reduced after the oral administration of sake yeast compared with the vehicle. The NREM sleep amounts at 3 h and 6 h significantly increased after the administration of sake yeast compared with the vehicle. The REM amount at 6 h significantly increased after the administration of sake yeast compared with the vehicle, but not at 3 h. The previous study suggested that the sleep-promoting effects of sake yeast could be referred from the activating effect of adenosine A_2A_ receptor (A_2A_R). In summary, the sake yeast is an A_2A_R agonist and may induce sleep due to its stress-reducing and anti-anxiety properties. Further verification of the involvement of adenosine in the pathophysiology of insomnia is needed.

## Introduction

Sleep plays an essential role in maintaining the homeostatic regulation. As sleep deprivation continues, homeostatic mechanism supplements the essential amount of sleep needed by promoting the recovery sleep with longer duration and deeper intensity. It is well-known that several endogenous molecules associated with the sleep regulation accumulate in the brain during the waking period. Moreover, these molecules in a certain concentration promote the transition from wakefulness to sleep state^1^. For example, adenosine, which is a potent endogenous sleep-promoting neuromodulator, accumulates in the basal forebrain and cortex during prolonged wakefulness or sleep deprivation^2,3^.

In modern society, the open access to the internet, hobbies, entertainment and exposure to the artificial illumination could induce insomnia, which from a public health perspective could be harmful to the health of people^4^. Insomnia according to its duration can be divided into acute or chronic insomnia. Acute insomnia was recognized in the International Classification of Disease (ICD) since 1977, in 1979 by the American Sleep Disorders Association and Diagnostic and in 1987 by the Statistical Manual of Mental Disorders (DSM)^5^. There are several causes of acute insomnia, including a change in environment, such as a new sleep environment, jet lag, clock change due to the exogenous factors, and the existence of acute physical symptoms, namely pain, urinary frequency, cough, and nasal congestion. Therefore, the difficulty in initiating or maintaining sleep is attributable to a stressor associated with physiological, psychological, physical, and environmental factors. In the context, acute insomnia should be treated by identifying and then resolving the underlying causes and sometimes with also a safe and effective medical treatment in order to prevent the progression to chronic insomnia^6^.

Medical treatment for insomnia includes the use of pharmacotherapy, such as the use of traditional benzodiazepines, nonbenzodiazepine sedative-hypnotics, melatonin receptor agonists, and orexin receptor antagonists, which were approved by the US Food and Drug Administration (FDA). However, there are well-known associated risks, including tolerance, addiction, abuse and paradoxical reactions including aggression, violence, and impulsivity, specially associated with the use of benzodiazepine but also in same degree to the nonbenzodiazepine^7–9^. Moreover, the use of benzodiazepine and nonbenzodiazepine is also associated with an increased risk for falls, bone fractures and cognitive dysfunction in old elderly^10–12^. Therefore, it is demanding to consider alternatives to these traditional pharmacotherapies for the reduction of harm.

Recent studies have shown that several natural compounds focused on the activation of adenosine receptor (AR) could induce the sleep-promoting effects^13^. Nakamura et al. showed that sake yeast is a dose-dependent adenosine A_2A_ receptor (A_2A_R) agonist that induces the enhanced non-rapid eye movement (NREM) sleep, possibly by accumulation of S-adenosylmethionine or methylthioadenosine, during the arousal phase by in mice^14^. Similarly, Monoi et al. demonstrated that sake yeast also improved the sleep quality in humans^15^. Since the sleep effects of sake yeast have already been demonstrated in previous studies, the novelty of the current study focused on investigating the sleep-inducing effects of sake yeast on acute insomnia induced, (1) during the sleep phase (dark period) in mice and (2) under a novel environment that disrupts sleep.

In the current study, the effects of sake yeast that is an adenosine analog was evaluated in the sleep/wake stages, locomotor activity, and core body temperature under a stressful environment as an acute insomnia model during sleep phase by using a sleep electroencephalography (EEG), an electromyography (EMG), and a telemetry implant in mice.

## Results

### The results of the preliminary experiment in active periods

After the oral administration of the vehicle (0.5% methylcellulose) and the sake yeast (at 300 mg/kg and 400 mg/kg) at the onset of dark period [*Zeitgeber* Time (ZT) 12], each mouse was replaced in habituated cage. Although significant differences were found [repeated multi-way analysis of variance (ANOVA), compound × time; F (10, 40) = 2.85, *p-value* = 0.009 and F (10, 40) = 3.18, *p-value* = 0.004, respectively] in the time course of wake and NREM sleep among the vehicle and the two doses tested of sake yeast, no clear tendency in drug effects were noticed (Supplementary Fig. S1A). The cumulative amounts of wake and NREM sleep were then displayed. Although no significant difference was found in the amounts of sleep/wake for overall 6 h (ZT12–18, dark period) after oral administration (*p-value* > 0.05), a dose-dependent wake reduction and NREM sleep enhancement were found after 4 h (ZT12–16) (Supplementary Fig. S1B,C). Moreover, a cumulative wake reduction (vehicle versus 400 mg/kg of sake yeast, *p-value* = 0.011) and a NREM sleep enhancement (vehicle versus 400 mg/kg of sake yeast, *p-value* = 0.006) at 4 h after sake yeast 400 mg/kg administrations were found to be statistically significant (Supplementary Fig. S1B). Additionally, 400 mg/kg of sake yeast administrations reduced locomotor activity and core body temperature for 4 h and 6 h after oral administration, however these effects were not statistically significant (*p-value* > 0.05, Supplementary Fig. S2).

### Sleep/wake stage analyses in new cages in resting periods

**Figure 1:**
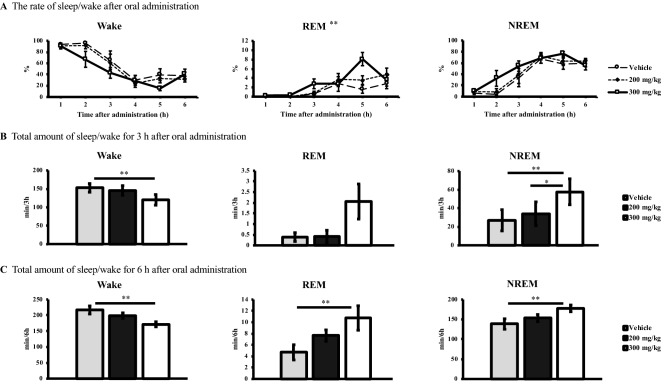
(**A**) The rate in percentage (%) of wake, REM sleep, and NREM sleep for 6 h (ZT2–8, light period) in a new cage after oral administration of vehicle and 200 mg/kg and 300 mg/kg of sake yeast with a 1 h interval. (**B**) Each total amount in minutes (min) of wake, REM sleep, and NREM sleep for 3 h (ZT2–5, light period) in a new cage after oral administration of vehicle and 200 mg/kg and 300 mg/kg of sake yeast. (**C**) Each total amount in minutes (min) of wake, REM sleep, and NREM sleep for 6 h (ZT2–8, light period) in a new cage after oral administration of vehicle and 200 mg/kg and 300 mg/kg of sake yeast. Error bars denote standard deviation of the mean. REM, rapid eye movement; NREM, non-rapid eye movement; ZT, *Zeitgeber* Time. * *p-value* < 0.05, ** *p-value* < 0.01.

Next, we focused on the effects of sake yeast under the stressful environment inducing the acute insomnia in a new cage. We assessed the time course of wake, REM sleep, and NREM sleep up to 6 h (ZT2–8, light period) after oral administration of the vehicle and 200 mg/kg and 300 mg/kg of sake yeast under the condition of acute insomnia (Fig. 1A). We found a statistically significant difference in the time course of REM sleep among the vehicle and sake yeast group through repeated multi-way ANOVA [compound × time; F (10, 50) = 3.52, *p-value* = 0.001], whereas the differences in the time course of wake and NREM sleep were not statistically significant (*p-value* > 0.05; Fig. 1A).

Moreover, the effects of sake yeast were then investigated for 3 h and 6 h after its administration on wake, REM sleep, and NREM sleep. We found dose-dependent significant differences in the total amounts of each sleep/wake stage for both of 3 h and 6 h, among the vehicle and 200 mg/kg and 300 mg/kg of sake yeast through repeated one-way ANOVA [at 3 h; wake: F (2, 10) = 9.46, *p-value* = 0.005 and NREM sleep: F (2, 10) = 9.64, *p-value* = 0.005, at 6 h; wake: F (2, 10) = 9.84, *p-value* = 0.004, REM sleep: F (2, 10) = 12.5, *p-value* = 0.002, and NREM sleep: F (2, 10) = 8.97, *p-value* = 0.006; Fig. 1B,C]. A post hoc analysis showed that at 3 h and 6 h after the administration of 300 mg/kg of sake yeast the amounts of wake was significantly reduced compared with those in the vehicle, respectively (*p-value* = 0.006 and *p-value* = 0.004, respectively). Regarding the effects on REM sleep, although no significant difference was found at 3 h, we showed the significant increment of the amount in the 300 mg/kg of sake yeast compared with that in vehicle at 6 h (*p-value* = 0.002). The amounts of NREM sleep at 3 h and 6 h after the administration of 300 mg/kg of sake yeast were significantly increased compared with those in the vehicle, respectively (*p-value* = 0.006 and *p-value* = 0.006, respectively). In addition, a significant difference in the amount of NREM sleep between 200 mg/kg and 300 mg/kg of sake yeast at 3 h was also demonstrated (*p-value* = 0.027).

### Locomotor activity and core body temperature

**Figure 2:**
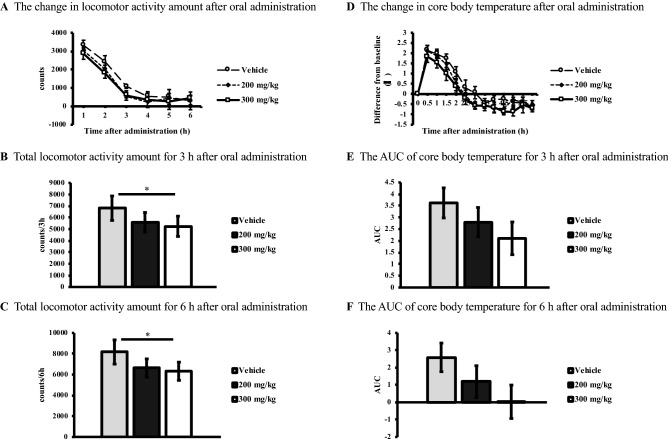
(**A**) Each locomotor activity amount (counts) for 6 h (ZT2–8, light period) in a new cage after oral administration of vehicle and 200 mg/kg and 300 mg/kg of sake yeast plotted with a 1 h interval. (**B**) Total locomotor activity amount (counts) for 3 h (ZT2–5, light period) in a new cage after oral administration of vehicle and 200 mg/kg and 300 mg/kg of sake yeast. (**C**) Total locomotor activity amount (counts) for 6 h (ZT2–8, light period) in a new cage after oral administration of vehicle and 200 mg/kg and 300 mg/kg of sake yeast. (**D**) The difference of core body temperature (°C) between before (baseline) and after oral administration of vehicle and 200 mg/kg and 300 mg/kg of sake yeast for 6 h (ZT2–8, light period) plotted with a 0.5 h interval. (**E**) The AUC of core body temperature for vehicle and 200 mg/kg and 300 mg/kg of sake yeast for 3 h (ZT2–5, light period). (**F**) The AUC of core body temperature for vehicle and 200 mg/kg and 300 mg/kg of sake yeast for 6 h (ZT2–8, light period). Error bars denote standard deviation of the mean. REM, rapid eye movement; NREM, non-rapid eye movement; ZT, *Zeitgeber* Time; AUC, area under the curve. * *p-value* < 0.05, ** *p-value* < 0.01.

The effects for 6 h after oral administration of 200 mg/kg and 300 mg/kg of sake yeast on locomotor activity and core body temperature were also investigated. First, we assessed the time course of locomotor activity up to 6 h after the administration of vehicle and 200 mg/kg and 300 mg/kg of sake yeast (Fig. 2A). Significant differences were found in the cumulative amounts of locomotor activity for 3 h and 6 h among the vehicle and 200 mg/kg and 300 mg/kg of sake yeast, using a repeated one-way ANOVA [F (2, 16) = 4.17, *p-value* = 0.035 and F (2, 16) = 5.75, *p-value* = 0.013, respectively; Fig. 2B,C]. A post hoc analysis showed that locomotor activities at 3 h and 6 h were significantly reduced by the administration of 300 mg/kg sake yeast compared with those at the vehicle only, respectively (*p-value* = 0.043 and *p-value* = 0.018, respectively). The time course differences in core body temperature were also investigated from pre-administration (baseline) up to 6 h after the administration of vehicle and 200 mg/kg and 300 mg/kg of sake yeast (Fig. 2D). Although there was no statistically significant difference in core body temperature for 3 h and 6 h among vehicle and sake yeast group, we found that core body temperature tended to decrease in a dose-dependent manner [F (2, 16) = 3.19, *p-value* = 0.068 and F (2, 16) = 2.64, *p-value* = 0.102, respectively; Fig. 2E,F].

## Discussion

The effects of sake yeast were studied on the sleep/wake stage, locomotor activity, and core body temperature under the conditions that stimulated acute insomnia in the light period in mice. Sake yeast in a dose-dependent manner increased both REM and NREM sleep, reducing the waking during the first 6 h after the oral administration of sake yeast with a decrease of the locomotor activity and core body temperature in a new cage.

A previous study conducted by Nakamura et al. showed a dose-dependent wake reduction and NREM sleep increment with a decreased locomotor activity in 100 mg/kg, 200 mg/kg and 300 mg/kg of sake yeast compared with the vehicle^14^. Contrasting the results obtained from our two experiments with those of previous studies, our preliminary study demonstrated a limited sleep-promoting effect of sake yeast, and the results were not necessarily consistent. In our preliminary study, as above-described, significant effects on sleep/wake were only found with a cumulative wake reduction and NREM sleep increment at 4 h (ZT12–16) after sake yeast 400 mg/kg administrations compared with vehicle but without affecting the locomotor activity. Although sleep-promoting effects were associated with a tendency to hypothermia, other significant effects were not observed when sake yeast was administered during active periods. There are several possible reasons for these non-complementary results. The results of sleep-promoting effects of nutraceutical natural compounds are mild compared with those of synthetic drugs may be inconsistent depending on the experimental conditions^16,17^. In fact, our preliminary study had several small differences in the experimental conditions from a previous study. Although the strain of mice (C57BL/6), sex (male), and time of administration (ZT12) were similar, several other conditions, such as age (previous study: 8 weeks and our preliminary study: 18 weeks), room temperature (22 °C ± 1 °C and 23 °C ± 1 °C), recovery period after surgery for telemetry implant (7 days and 2 weeks), and method of measuring locomotor activity (infrared sensor and telemetry receiver) were substantially different. On the other hand, the results of our experiment performed under a validated acute insomnia model and the results of the previous study both showed the sleep-inducing effect of sake yeast in a dose-dependent manner^18^. The mice were placed in new cages immediately after administration of vehicle or sake yeast at ZT2 on the light period, being monitored the effects of sleep at 3 h and 6 h after its administration by reducing the dose of sake yeast (200 mg/kg and 300 mg/kg) compared with the dose used in the preliminary experiment. The reason for selecting these doses for a second set is that our preliminary experiment showed that sake yeast lowered core body temperature in a dose-dependent manner, especially transiently by up to − 0.23 °C from baseline after 400 mg/kg dose and by up to − 1.55 °C from baseline after 500 mg/kg dose without sleep-inducing effects (Supplementary Fig. S3). Consequently, it was confirmed that sake yeast could induce the increase in REM and NREM sleep, as well as to decreased locomotor activity in dose-dependent manner and being also able to decrease the core body temperature in stress-induced acute insomnia model during sleep phase.

Nakamura et al. showed that NREM sleep-promoting effect of sake yeast could be closely related to the receptor A_2A_R. This interpretation is based on results that demonstrate that sake yeast enhanced NREM sleep after the preadministration of A_1_R antagonist but not after the preadministration of A_2A_R antagonist^14^. In fact, it is widely known that adenosine plays an important role in the homeostatic sleep regulation^2,19^. Indeed, adenosine, a purine nucleoside consisted of a molecule of adenine attached to a ribose sugar molecule (ribofuranose) via a β-*N*_9_-glycosidic bond, is a neuromodulator that possesses several inhibitory mechanisms, being able to regulate the brain metabolism^20^. There are four AR subtypes, namely A_1_R, A_2A_R, A_2B_R, and A_3_R, being all G-protein-coupled receptors. A_1_R and A_2A_R are reported to be primarily associated with sleep regulation^21,22^, and thus adenosine promotes sleep by activating the A_1_R and A_2A_R^23^. In addition to sake yeast, there have been several basic researches focusing on the sleep-promoting effects of natural components that act as A_2A_R agonists^13,24–27^. Indeed, whereas there is a clear evidence that adenosine is involved in sleep regulation and sleep disorders, adenosinergic compounds are being rarely used in the sleep disorders treatment. The novelty of the current study is confirmation of the efficacy of sake yeast as an A_2A_R agonist in stress-induced acute insomnia model in mice. A previous study has shown that A_2A_R deficiency is associated with a profile of anxiety for a novel environment^28^. The stress-reducing or anti-anxiety effects of A_2A_R agonist, in addition to its sleep-regulating effects, may be involved in the sleep-promoting effects of sake yeast, which were not seen in the arousal phase but only under the acute insomnia model in the sleep phase in the current study. The involvement of adenosine in the pathophysiology of insomnia is of extreme interest and needs to be further investigated.

From a public health perspective, sleep deprivation could induce several adverse effects on the health, productivity, and performance^29^. Therefore, the individuals with sleep deprivation might have a decreased longevity and cerebral and physical dysfunctions. The sleep quality is often dependent on the external environment, such as ambient sound, light, and air quality^30^. The individuals who could not get enough sleep temporarily under these unfavorable situations might develop acute insomnia. In the current study, the efficacy of sake yeast was investigated in acute insomnia caused by the change in new environment in mice. In the clinical practice, acute insomnia is often treated with sedative hypnotics such as benzodiazepines, nonbenzodiazepines, melatonin receptor agonists, and orexin receptor antagonist to prevent the daytime impairment and the transition to chronic insomnia^6^. Nevertheless, all the benzodiazepine could induce the several and frequent side effects, including sedation, dizziness, weakness, and unsteadiness, physical dependence, and excessive sedation due to the negative interaction with barbiturates, anesthetics, and alcohol^8,9^. Thus, both medical staff and patients need to pay adequate attention to these adverse events caused by the pharmacotherapy.

In conclusion, this study demonstrated that sake yeast, through the activation of A_2A_R, promotes both REM and NREM sleep by modulating locomotor activity and thermoregulation not only through the sleep-inducing effects demonstrated in previous studies, but also under the condition of acute insomnia induced by a novel environment. In the future, it will be important to investigate the involvement of adenosine in the pathophysiology of insomnia.

## Methods

### Materials

The sake yeast powder administered in this study was provided by a manufacture^14^. Sake yeast was stored at 2–8 °C in the dark. For the control (vehicle) administered to the mice, 0.5% methylcellulose solution was used.

### Animals

Nine C57BL/6 male mice, aged 20 weeks (Jackson laboratory, Bar Harbor, ME, USA), were maintained on a 12-h light/dark cycle with room temperature at 23 °C ± 1 °C. Each mouse was housed in its own individual recording cage (22 cm × 16 cm × 12 cm). Food and water were available ad libitum. This study was approved by and was performed according to the Stanford University Administrative Panel on Laboratory Animal Care Guidelines (APLAC-21646). This study is reported in accordance with ARRIVE guidelines.

### Surgery for telemetry implant and headstage

As previously reported, the surgical procedures for telemetry and headstage were conducted in this study^31^. A telemetry implanting device (G2 E-Mitter; Mini Mitter OR, Oakmont, PA, USA) was implanted in the abdominal cavity of the mice under 3% isoflurane anesthesia in order to evaluate the locomotor activity and core body temperature. The mice were surgically intervened for EEG and EMG recordings using a headstage installed to the cable recorder. The four electrodes for the EEG were composed of stainless-steel screws, and two of them were screwed into the skull 1.5 mm lateral and 1.5 mm anterior to the bregma over the motor cortex. The other two electrodes were screwed 3 mm lateral and 1 mm anterior to the lambda over the visual cortex. Two Teflon-coated stainless-steel wires for the EMG were placed into the neck extensor muscles on mice both sides. The six electrodes were installed to one 2 × 3 pin header that was secured to the skull using dental cement. After the surgery, the mice were subcutaneously injected with an analgesic (carprofen, 3 mg/kg) and one antibiotic (enrofloxacin, 25 mg/kg), and then allowed to recover during 2 weeks before performing the experiments.

### Data collection

After 2 weeks of postoperative recovery phase, each mouse was moved to a specially modified experimental cage equipped with a low-torque slip-ring commutator (Biella Engineering, Irvine, CA, USA). The cages were placed in the recording chamber. The next day, the headstages of the mice were connected to the EEG/EMG recording cables, which were composed of the slip ring commutator through a 15–20 cm of lightweight 6-strand shielded signal cable (NMUF6/30-4046SJ; Cooner Wire, Chatsworth, CA, USA). The output of commutator was then connected to the amplifier. The mice could have freely access to food and water inside the cages. The temperature of experimental laboratory was maintained at 23 °C ± 1 °C. A 12-h light/dark cycle was performed throughout the experiment. After 1 week of habituation to the experiment environment, consecutive EEG/EMG, locomotor activity and core body temperature were recorded.

The EEG/EMG signals were acquired using a Glass Instrument model 12 (West Warwick, RI, USA). The EEG/EMG signals were digitally filtered (30 Hz Low Pass Filter for EEG; 10–100 Hz Band Pass Filter for EMG). The EEG signals were captured at 256 Hz, using a sleep recording system (Vital Recorder; Kissei Comtec Co. Ltd., Matsumoto, Japan). EEG signals were collected with ipsilateral bipolar electrodes, which were placed over motor and visual cortices, together with the bipolar EMG signals, which were employed for sleep scoring.

### Oral administration of sake yeast

In all experiments, sake yeast was dissolved in the same 0.5% methylcellulose solution as the vehicle. The prepared sake yeast solution and vehicle were administered to mice at a dose of 10 ml/kg each.

### Design for preliminary experiment and acute insomnia inducing experiment

**Figure 3:**
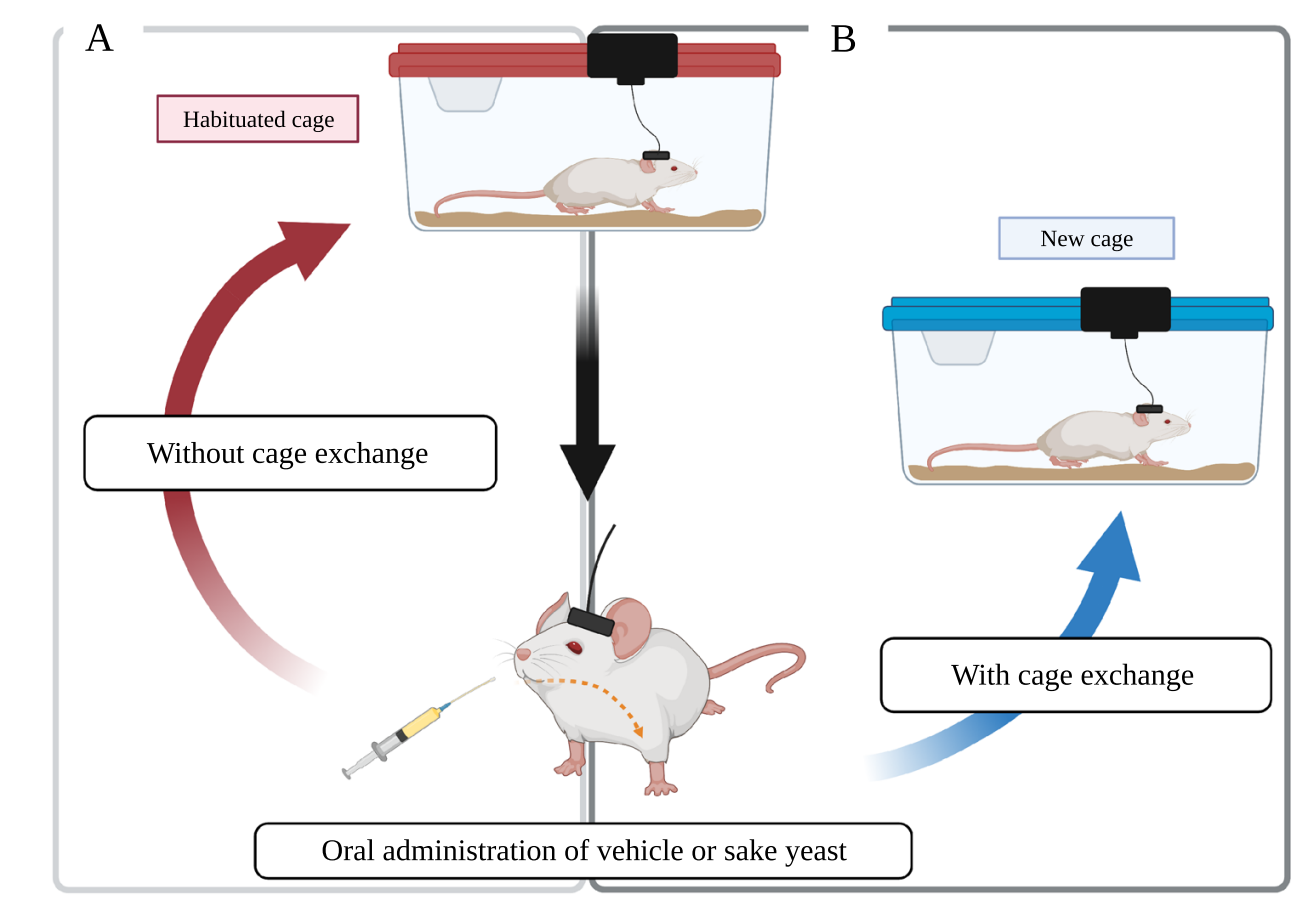
Procedure for the acute insomnia model. Each mouse was housed in its own individual habituated cage before oral administration. (**A**) In our preliminary experiment, each mouse was replaced in habituated cage (without cage exchange) immediately after oral administration of vehicle or sake yeast. (**B**) In the current study, each mouse was placed in a new, clean experimental cage supposing the condition of acute insomnia (with cage exchange) immediately after oral administration (Created with BioRender.com).

First, we conducted a preliminary experiment to examine the sleep-promoting effects of sake yeast using mice (six C57BL/6 male mice, aged 18 weeks) in habituated housing cages in order to reconfirm the results of previous studies. In our preliminary experiment, vehicle and 300 mg/kg and 400 mg/kg of sake yeast were orally administered at the onset of dark period (ZT12, arousal phase), with an interval of at least 48 h. After the administration, each mouse was immediately replaced to its original habituated cage (without cage exchange; Fig. 3A).

Next, sake yeast in two different doses (i.e., 200 mg/kg and 300 mg/kg) and the vehicle without sake yeast were orally administrated to different groups of mice from those used in the preliminary experiment (nine C57BL/6 male mice, aged 20 weeks) at ZT2 (light period) on different days, with an interval of at least 48 h. Immediately after the oral administration, each mouse was transferred from its habituated cage to a new, clean experimental cage to investigate the effect of sake yeast in a novel environment inducing the condition of acute insomnia (with cage exchange; Fig. 3B). We did not stress the mice in any way, except for cage exchange. This model of acute insomnia with cage exchange has already been validated in our previous experiment^18^.

### Sleep recording

As previously reported, the sleep/wake stages based on EEG and EMG signals in 10-s epochs were visually analyzed using the standard criteria^31^. In addition, 50% or more of a specific state in each epoch was defined to score the epoch. Wakefulness was determined as desynchronized, low-amplitude, and mixed-frequency (> 4 Hz) EEG associated with a high EMG activity. The rhythmic theta/alpha (7–9 Hz) wave was also detected. NREM sleep was determined as synchronized, high-amplitude, and low-frequency (0.25–4 Hz) EEG with a low EMG activity. Alike wakefulness, the REM sleep was also determined as desynchronized, low-amplitude, and mixed-frequency EEG. However, the EMG activity during REM sleep was lower than that during NREM sleep. During REM sleep, some muscle twitching were detected in the EMG trace. Basically, rhythmic theta/alpha (7–9 Hz) waves with low EMG activity are dominant. Sleep state changes were considered when at least one 10-s epoch was detected as appearing a different sleep stage, being the duration of the state episode defined as the duration of a continuous single state episode. A single investigator (S.N.) who was blinded to the animal information was responsible to record all the scoring.

### Sleep/wake data analysis

The rate in percentage (%) obtained for wake, REM sleep, and NREM sleep for 6 h (ZT2–8, light period) after oral administration of sake yeast and vehicle was calculated and plotted with a 1 h interval. In addition, the cumulative amount in minutes (min) for 6 h after administration’s sake yeast and vehicle were also calculated and plotted with an interval of 1 h.

### Locomotor activity and core body temperature data analysis

As previously reported, locomotor activity and core body temperature data were measured using the telemetry receiver (Series 4000, Mini Mitter) that transmitted the signals for these physiological data every 1 min, being analyzed using the Vital View software (Mini Mitter, OR)^31^. The mean value, as well as cumulative amount of locomotor activity (counts/h) for 6 h after the administration of sake yeast and vehicle were calculated and plotted with a 1 h interval. Additionally, the 0.5 hourly mean value of core body temperature (°C) after the administration of sake yeast and vehicle was also calculated. Then, the difference of temperature between before (baseline) and after the administration was calculated and plotted with a 0.5 h interval for 6 h (ZT2–8, light period) to normalize the variability of baseline. Moreover, the area under the curve (AUC) was also calculated 6 h after administration of sake yeast and vehicle.

### Statistical analysis

All the results presented in this study were expressed as the mean ± standard deviation of the mean. All the statistical analyses were conducted using the SPSS version 22 software (IBM Corp., Armonk, NY, USA). The total amounts of sleep/wake, locomotor activity, and core body temperature changes were analyzed using repeated one-way ANOVA, followed by Bonferroni’s test for multiple comparisons. The time course changes in the amounts of sleep/wake, locomotor activity, and core body temperature were also analyzed using repeated two-way ANOVA (compound group [vehicle, 200 mg/kg, and 300 mg/kg], time, and compound × time). The level of statistical significance was set at *p-value* < 0.05.

## Supplementary Information

Supplementary Figure 1.

Supplementary Figure 2.

Supplementary Figure 3.

Supplementary Legends.

## Abbreviations

REM: Rapid eye movement
NREM: Non-rapid eye movement
AR: Adenosine receptor
A_1_R: Adenosine A_1_ receptor
A_2A_R: Adenosine A_2A_ receptor
A_2B_R: Adenosine A_2B_ receptor
A_3_R: Adenosine A_3_ receptor
ZT: *Zeitgeber* Time
