# Supplementary material for: Sake yeast induces the sleep-promoting effects under the stress-induced acute insomnia in mice
Source: Sci Rep. 2021 Oct 21;11:20816. doi: 10.1038/s41598-021-00271-0 (PMC8531297; doi:10.1038/s41598-021-00271-0)
Supplement: Supplementary file 1 — Supplementary Figure 1. [file 41598_2021_271_MOESM1_ESM.pdf]

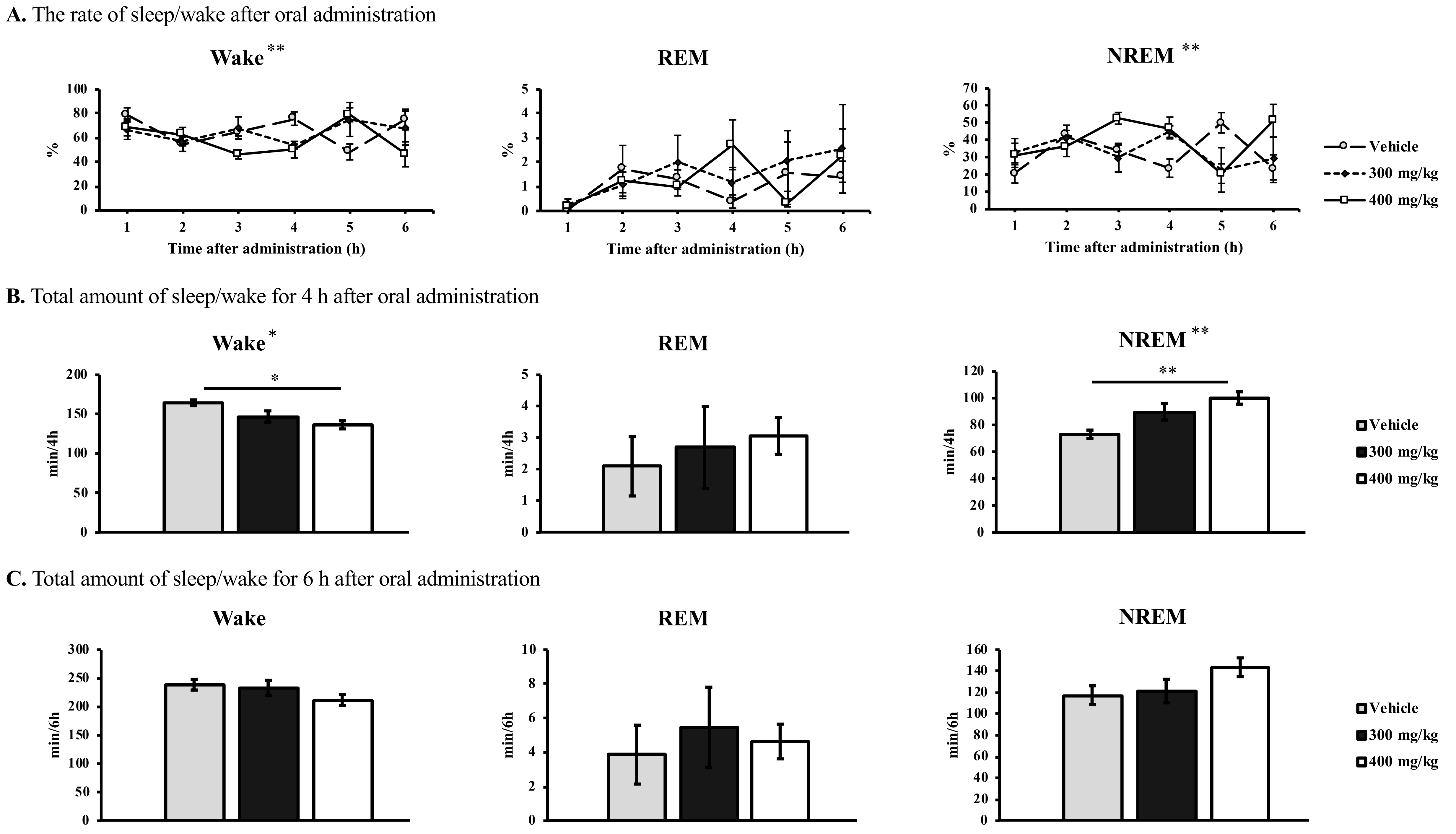
A. The rate of sleep/wake after oral administration
Wake **
REM
NREM **
%
Time after administration (h)
Vehicle
300 mg/kg
400 mg/kg
B. Total amount of sleep/wake for 4 h after oral administration
Wake *
REM
NREM **
min/4h
*
**
Vehicle
300 mg/kg
400 mg/kg
C. Total amount of sleep/wake for 6 h after oral administration
Wake
REM
NREM
min/6h
Vehicle
300 mg/kg
400 mg/kg
