# Supplementary material for: Sake yeast induces the sleep-promoting effects under the stress-induced acute insomnia in mice
Source: Sci Rep. 2021 Oct 21;11:20816. doi: 10.1038/s41598-021-00271-0 (PMC8531297; doi:10.1038/s41598-021-00271-0)

A. The change in locomotor activity amount after oral administration

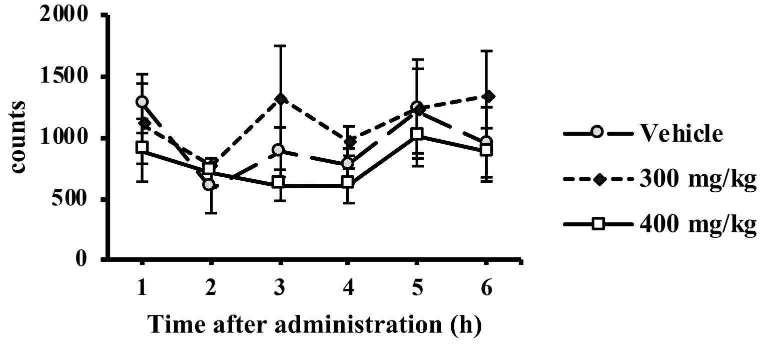

B. Total locomotor activity amount for 4 h after oral administration

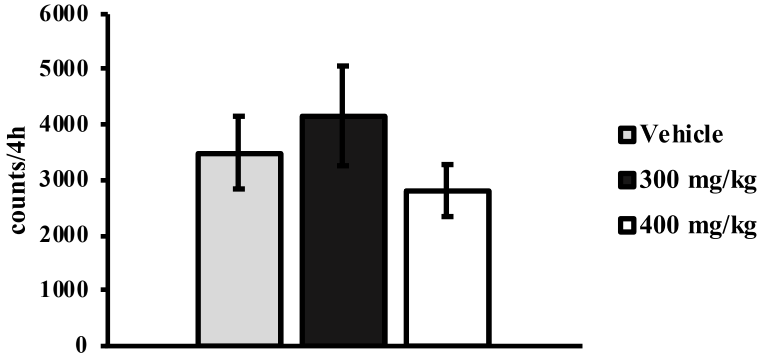

C. Total amount of wake/sleep for 6 h after oral administration

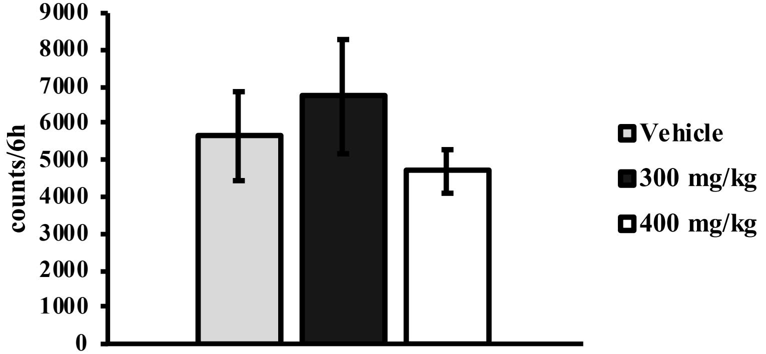

D. The change in core body temperature after oral administration

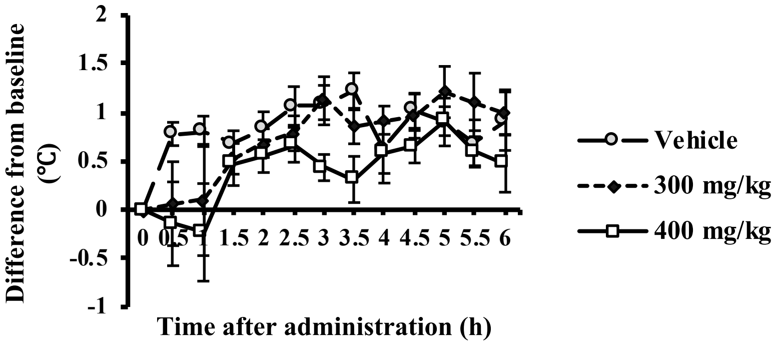

E. The AUC of core body temperature for 4 h after oral administration

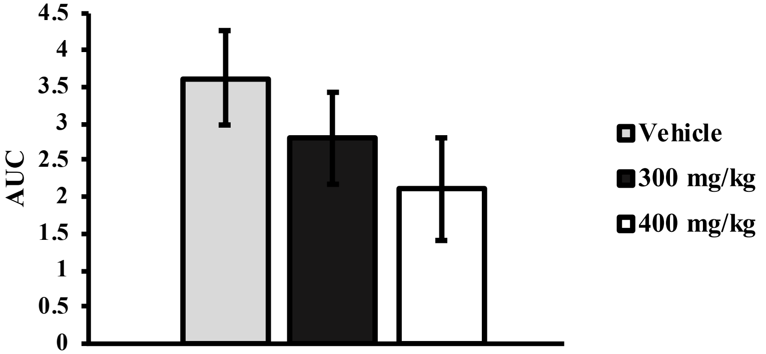

F. The AUC of core body temperature for 6 h after oral administration

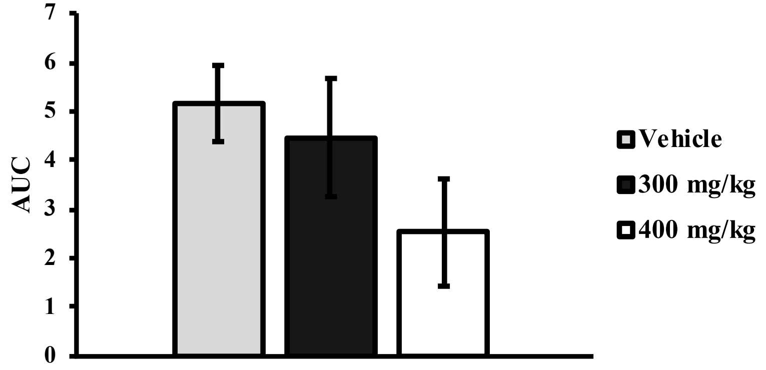

Supplement: Supplementary file 2 — Supplementary Figure 2. [file 41598_2021_271_MOESM2_ESM.pdf]
