# Supplementary figures and images for: Sake yeast induces the sleep-promoting effects under the stress-induced acute insomnia in mice
Source: Sci Rep. 2021 Oct 21;11:20816. doi: 10.1038/s41598-021-00271-0 (PMC8531297; doi:10.1038/s41598-021-00271-0)

## The change in core body temperature after oral administration

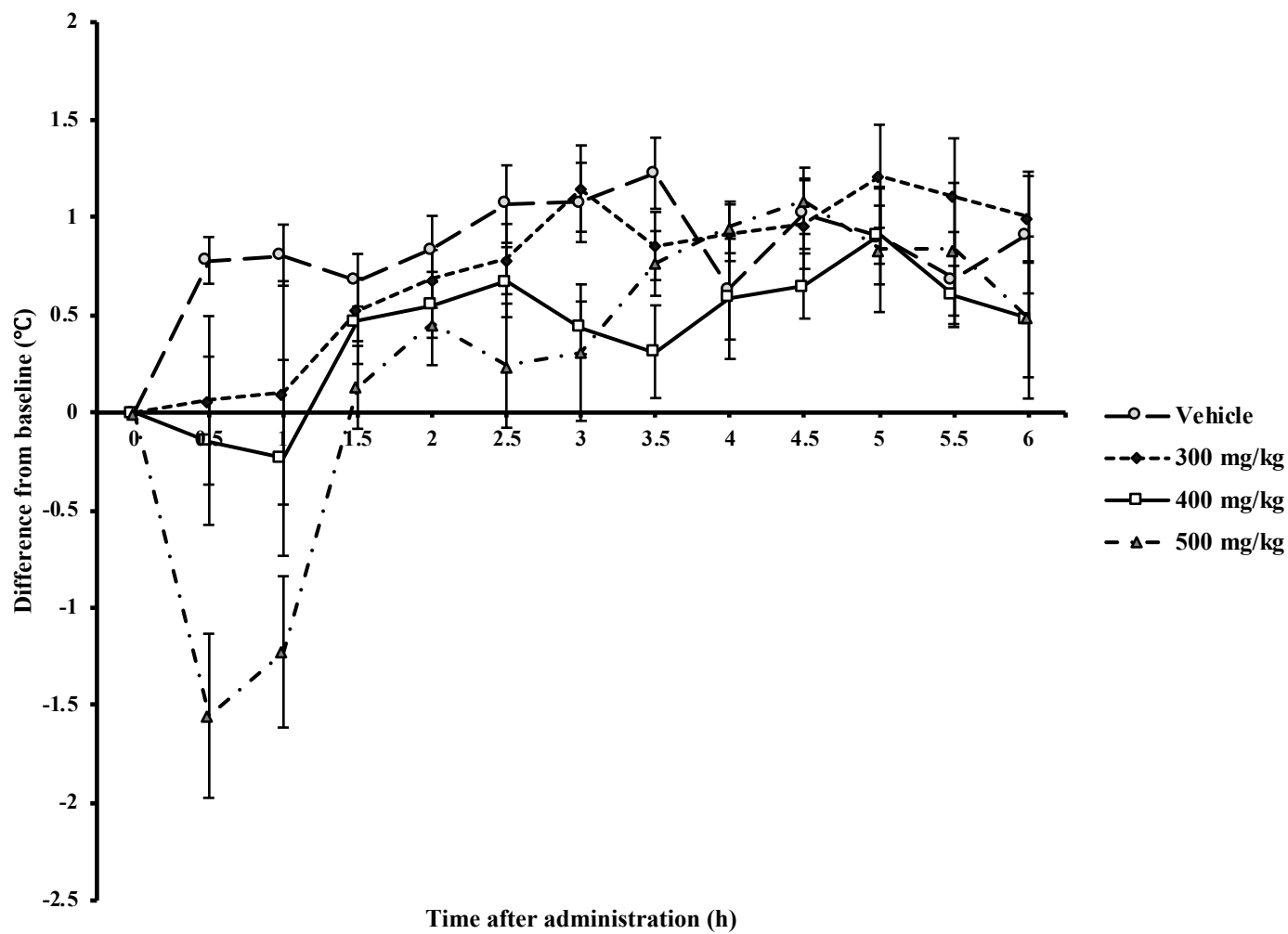

Supplement: Supplementary file 3 — Supplementary Figure 3. [file 41598_2021_271_MOESM3_ESM.pdf]
