# Supplementary material for: Sake yeast induces the sleep-promoting effects under the stress-induced acute insomnia in mice
Source: Sci Rep. 2021 Oct 21;11:20816. doi: 10.1038/s41598-021-00271-0 (PMC8531297; doi:10.1038/s41598-021-00271-0)
Supplement: Supplementary file 4 — Supplementary Legends. [file 41598_2021_271_MOESM4_ESM.docx]

**Supplementary Figure captions**

**Supplementary Figure 1** (**A**) The rate in percentage (%) of wake, REM sleep, and NREM sleep for 6 h (ZT12–18, dark period, arousal phase) after oral administration of vehicle and 300 mg/kg and 400 mg/kg of sake yeast with a 1 h interval. (**B**) Each total amount in minutes (min) of wake, REM sleep, and NREM sleep for 4 h (ZT12–16, dark period) after oral administration of vehicle and 300 mg/kg and 400 mg/kg of sake yeast. Error bars denote standard deviation of the mean. (**C**) Each total amount in minutes (min) of wake, REM sleep, and NREM sleep for 6 h (ZT12–18, dark period) after oral administration of vehicle and 300 mg/kg and 400 mg/kg of sake yeast. Error bars denote standard deviation of the mean. REM, rapid eye movement; NREM, non-rapid eye movement; ZT, *Zeitgeber* Time. * *p-value* < 0.05, ** *p-value* < 0.01

**Supplementary Figure 2** (**A**) Each locomotor activity amount (counts) for 6 h (ZT12–18, dark period, arousal phase) after oral administration of vehicle and 300 mg/kg and 400 mg/kg of sake yeast plotted with a 1 h interval. (**B**) Total locomotor activity amount (counts) for 4 h (ZT12–16, dark period) after oral administration of vehicle and 300 mg/kg and 400 mg/kg of sake yeast. (**C**) Total locomotor activity amount (counts) for 6 h (ZT12–18, dark period) after oral administration of vehicle and 300 mg/kg and 400 mg/kg of sake yeast. (**D**) The difference of core body temperature (°C) between before (baseline) and after oral administration of vehicle and 300 mg/kg and 400 mg/kg of sake yeast for 6 h (ZT12–18, dark period) plotted with a 0.5 h interval. (**E**) The AUC of core body temperature for vehicle and 300 mg/kg and 400 mg/kg of sake yeast for 4 h (ZT12–16, dark period). (**F**) The AUC of core body temperature for vehicle and 300 mg/kg and 400 mg/kg of sake yeast for 6 h (ZT12–18, dark period). Error bars denote standard deviation of the mean. REM, rapid eye movement; NREM, non-rapid eye movement; ZT, *Zeitgeber* Time; AUC, area under the curve. * *p-value* < 0.05, ** *p-value* < 0.01

**Supplementary Figure 3** The difference of core body temperature (°C) between before (baseline) and after oral administration of vehicle and 300 mg/kg, 400 mg/kg, and 500 mg/kg of sake yeast for 6 h (ZT12–18, dark period) plotted with a 0.5 h interval. Error bars denote standard deviation of the mean. ZT, *Zeitgeber* Time.
